# Immune Profile in COVID-19: Unveiling T_R3-56_ Cells in SARS-CoV-2 Infection

**DOI:** 10.3390/ijms251910465

**Published:** 2024-09-28

**Authors:** Flavia Carriero, Valentina Rubino, Monica Gelzo, Giulia Scalia, Maddalena Raia, Massimo Ciccozzi, Ivan Gentile, Biagio Pinchera, Giuseppe Castaldo, Giuseppina Ruggiero, Giuseppe Terrazzano

**Affiliations:** 1 Dipartimento di Scienze della Salute, Università degli Studi della Basilicata, 85100 Potenza, Italy; flavia.carriero@unibas.it; 2 Dipartimento di Scienze Mediche Traslazionali, Università di Napoli “Federico II”, 80131 Naples, Italy; valentina.rubino@unina.it (V.R.); giuseppina.ruggiero@unina.it (G.R.); 3 CEINGE-Biotecnologie Avanzate Franco Salvatore, 80131 Naples, Italy; monica.gelzo@unina.it (M.G.); scalia@ceinge.unina.it (G.S.); raia@ceinge.unina.it (M.R.); giuseppe.castaldo@unina.it (G.C.); 4 Dipartimento di Medicina Molecolare e Biotecnologie Mediche, Università di Napoli Federico II, 80131 Naples, Italy; 5 Unità di Epidemiologia e Statistica Medica, Università Campus Biomedico, 00128 Rome, Italy; m.ciccozzi@unicampus.it; 6 Dipartimento di Medicina Clinica e Chirurgia, Università di Napoli Federico II, 80131 Naples, Italy; ivan.gentile@unina.it (I.G.); biagio.pinchera@unina.it (B.P.)

**Keywords:** SARS-CoV-2, Severe acute respiratory syndrome coronavirus 2, COVID-19, COronaVIrus Disease 2019, immune regulation, immune regulatory cell phenotypes, T_R3-56_

## Abstract

The emergence of COronaVIrus Disease 2019 (COVID-19), caused by severe acute respiratory syndrome coronavirus 2 (SARS-CoV-2), presented a global health challenge since its identification in December 2019. With clinical manifestations ranging from mild respiratory symptoms to severe multi-organ dysfunction, COVID-19 continues to affect populations worldwide. The complex interactions between SARS-CoV-2 variants and the human immune system are crucial for developing effective therapies, vaccines, and preventive measures. Understanding these immune responses highlights the intricate nature of COVID-19 pathogenesis. This retrospective study analyzed, by flow cytometry approach, a cohort of patients infected with SARS-CoV-2 during the initial pandemic waves from 2020 to 2021. It focused on untreated individuals at the time of hospital admission and examined the presence of T_R3-56_ cells in their immune profiles during the anti-viral immune response. Our findings provide additional insights into the complex immunological dynamics of SARS-CoV-2 infection and highlight the potential role of T_R3-56_ cells as crucial components of the immune response. We suggest that T_R3-56_ cells could serve as valuable biomarkers for identifying more severe cases of COVID-19, aiding in the assessment and management of the disease.

## 1. Introduction

The emergence of the severe acute respiratory syndrome coronavirus 2 (SARS-CoV-2) presented a global health challenge [1,2], exhibiting a spectrum of clinical manifestations ranging from mild respiratory symptoms to severe pneumonia and multi-organ dysfunction, COronaVIrus Disease 2019 (COVID-19) [3,4,5]. The intricate interplay between the new SARS-CoV-2 variants and the human immune system remains crucial for developing effective diagnostic biomarkers, therapeutic interventions, vaccines, and preventive strategies [5,6,7,8].

Subsequent to viral entry [1,2,3,4,5], a series of immunological events are set, activating innate and adaptive immune responses aimed at controlling viral replication and eliminating infected cells [9,10,11,12,13,14,15,16,17]. As of mid-2024, COVID-19 persists as a global health challenge, and advancements in vaccines and ongoing public health efforts are essential to managing its impact in the future.

Literature highlighted various immune responses during SARS-CoV-2 infection and described a broad involvement of the immune system in contrasting this infection, offering new insights for a comprehensive understanding of the processes and mechanisms of susceptibility and resistance to the infection. Of particular relevance is the phenomenon known as “cytokine storm,” characterized by an excessive release of pro-inflammatory cytokines, which can lead to endothelial, acute respiratory distress syndrome (ARDS), and multi-organ failure in COVID-19 [9,10,11,12,13,14,15].

Natural killer (NK) cells play a crucial role in the innate immune response against SARS-CoV-2 contributing to early antiviral defence [9,10,11,12,16]. CD8+ T cells, also called Cytotoxic T lymphocytes (CTLs), directly eliminate virus-infected cells [11,12,13,14,17]. CD4+ T cells, also known as T helper (Th) cells, play a crucial role in coordinating immune responses [17,18]. Th1 cells are pivotal in the adaptive immune response against SARS-CoV-2, since they activate the immune response by producing cytokines such as interferon-gamma (IFN-γ), which boosts macrophage phagocytic activity and antigen presentation and supports the differentiation in CTLs [9,10,11,12,13,18].

Several cytokines are described to be mainly involved in COVID-19 [11,12,19,20]. In this regard, tumor necrosis factor-alpha (TNF-α) contributes to the cytokine storm [9,10,11,12,13,15,21]. Interleukin (IL)-6 drives the inflammatory response, with elevated levels linked to severe COVID-19 and complications such as ARDS [9,10,11,12,13,22,23]. While IL-10 helps to mitigate excessive inflammation [24], its role in the immune response to COVID-19 is still controversial [9,10,11,12,13]. Th17 cells may foster the immune response against SARS-CoV-2 by producing IL-17 and other cytokines that recruit neutrophils and help in combating viral infection to preserve mucosal integrity [9,10,11,12,13,25,26]. IL-17 can also amplify inflammatory responses by inducing the production of other pro-inflammatory cytokines (e.g., IL-6, TNF-α) [25,26].

B lymphocytes (B cells) and antibodies are critical components of the immune response against COVID-19. When B cells encounter the SARS-CoV-2 virus, they produce virus-specific antibodies that neutralize the virus and prevent it from infecting cells. No significant association was found between mortality and IgG or IgM seroconversion or antibody concentrations. Patients with severe COVID-19 tend to develop an early and robust humoral immune response, characterized by the production of SARS-CoV-2-specific IgG antibodies [9,10,11,12,27,28,29].

Dysregulated immune responses, marked by hyperinflammation and cytokine storm, were implicated in the pathogenesis of severe COVID-19, leading to tissue damage, vascular dysfunction, and multi-organ failure. Conversely, an effective and coordinated immune response involving both innate and adaptive immune mechanisms is crucial for viral clearance and resolution of infection [1,2,3,4,5,6,7,30,31,32]. In this regard, the immune system comprises a complex network of cells and molecules that safeguard the host against pathogens, including viruses. Within this intricate system, several subsets of immune cells play a pivotal role in orchestrating immune responses [33,34,35,36].

Among lymphocytes, regulatory T cells (Tregs) garnered significant attention for their ability to modulate immune function and maintain tolerance to self-antigens while preventing excessive immune responses to foreign invaders [34,35,36]. Dysregulation of Tregs can lead to autoimmune diseases or immune suppression, affecting overall immune function [34,35,36].

Several T lymphocyte subpopulations co-expressing CD3 and CD56 molecules were identified [37,38,39,40,41,42,43,44,45,46,47,48,49,50,51,52,53,54,55,56,57,58,59,60,61]. The CD3+ CD56+ T cell subtype is a distinct group displaying both T cell (i.e., CD3) and NK (i.e., CD56) characteristics [33,37,38,39,40,41,42,43,44,45,46,47,48,49,50,51,52,53,54,55,56]. Within this group, natural killer T (NKT) cells are notable for bridging innate and adaptive immunity by recognizing lipid antigens presented by CD1d molecules and producing cytokines such as IFN-γ and IL-4 [37,38,39,40,41,42,43,44,45]. While NKT cells are well-studied, the roles of other CD3+ CD56+ T cell subtypes are unclear, although they are involved in cytotoxic activity, cytokine production, and possibly in immune regulation and disease mechanisms [45,46,47,48,49,50,51,52,53,54]. These cells are elevated in conditions such as solid tumors, non-alcoholic fatty liver disease, autoimmune disorders, and haematological malignancies, where they may contribute to disease pathology [45,46,47,48,49,50,51,52,53] and are often cytotoxic effectors [54,55,56].

Recently, we described a regulatory role for a subtype of CD3+ CD56+ T cells, defined as T_R3-56_ [57,58,59,60,61]. These cells exhibit a unique metabolic phenotype, primarily relying on oxidative phosphorylation, and possess a distinct transcriptomic profile compared to NK, NKT, CD3+CD56-, and CD8+ T cells. Our original studies focused on type 1 diabetes (T1D), revealing that T1D patients had significantly reduced T_R3-56_ cells, correlating with increased CTL activation and disease severity [57]. Lower frequencies of T_R3-56_ cells were associated with decreased β-cell function and diabetic ketoacidosis, and in our T1D cohorts, T_R3-56_ cells were shown to suppress CTL functions in vitro by reducing intracellular reactive oxygen species, with their suppressive function and phenotype altered in T1D children. Our findings suggest T_R3-56_ cells play a regulatory role in modulating CTLs and could serve as a biomarker for monitoring immunological self-tolerance in T1D. In myelodysplastic syndromes (MDS), T_R3-56_ cells inversely correlated with cytotoxic T cell activation, suggesting a regulatory role also in bone marrow [58,59]. Similarly, increased TR3-56 cells, proportional to Tregs, may contribute to immune escape in chronic lymphocytic leukaemia (CLL) [61].

Therefore, we proposed the role of T_R3-56_ lymphocytes as a new cellular candidate in the immune regulation landscape [33].

Given the growing interest in the role of all CD3+ CD56+ T cell subtypes, it is relevant to further investigate their involvement in various disease models, particularly in viral infections, as recently highlighted in COVID-19 [62,63].

This retrospective study aims to explore the role of T_R3-56_ cells in SARS-CoV-2 infection during the first and second waves of the pandemic (March 2020–April 2021). Based on prior analyses of an established patient cohort [20,64], classified according to World Health Organization (WHO) criteria [65], the study specifically focused on individuals who never received any treatment before hospitalization. While SARS-CoV-2 serves as an initial model for this investigation, the ultimate goal is to further advance the study of T_R3-56_ cells in viral infections.

## 2. Results

### 2.1. The Immune Asset in the Patients Stratified on the Severity of COVID Disease

We categorized COVID-19 patients into three groups: Group 1 (WHO 3), Group 2 (WHO 4), and Group 3 (WHO 5, 6, and 7), following our previously described stratification [20] based on WHO criteria [65]. However, in the current study, we analyzed data exclusively from hospitalized patients who never received therapy or anti-SARS-CoV-2 vaccination prior to admission (see Section 4).

**Figure 1:**
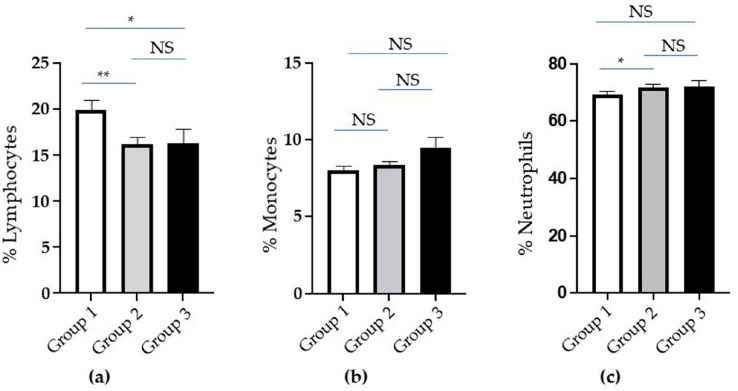
Analysis of white blood cells (WBCs) in groups of COronaVIrus Disease 2019 (COVID-19) patients based on increasing severity. (**a**) The percentage of whole lymphocytes, (**b**) monocytes, and (**c**) neutrophils in Groups 1 (white columns), 2 (grey columns), and 3 (black columns) of patients. Standard error (SE) bars are reported at the top of the columns. Statistical analysis (*Mann–Whitney test*) is reported: *p* ≤ 0.05 (*); *p* ≤ 0.005 (**); not significant (NS).

Based on the evaluation of the percentage of whole white blood cells (WBCs), the lymphocytes were significantly reduced in the Groups 2 and 3 with more severe clinical conditions (Figure 1a). No statistically significant differences are observed in the monocyte population (Figure 1b). Finally, neutrophils slightly increased in Group 2 compared to Group 1 (Figure 1c).

**Figure 2:**
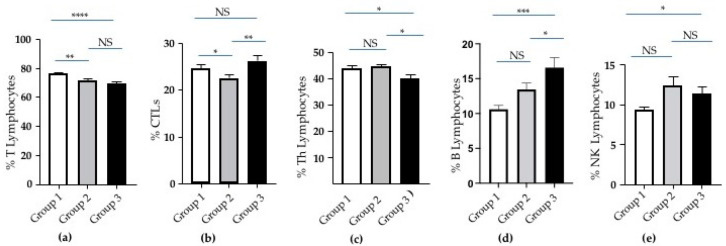
Analysis of lymphocyte subtypes in groups of COVID-19 patients based on increasing severity. (**a**) The percentage of T, (**b**) cytotoxic T cells (CTLs), (**c**) T helper (Th), (**d**) B, and (**e**) Natural Killer (NK) lymphocytes in Groups 1 (white columns), 2 (grey columns), and 3 (black columns) of patients. Standard error (SE) bars are reported at the top of the columns. Statistical analysis (*Mann–Whitney test*) is reported: *p* ≤ 0.05 (*); *p* ≤ 0.005 (**); *p* ≤0.0005 (***); *p* < 0.0001 (****); and not significant (NS).

Focusing on the lymphocyte population among the WBCs, we observed a reduction in the percentage of T cells in Groups 2 and 3 compared to Group 1 (Figure 2a). CTLs were significantly lower in Group 2 compared to Group 1 and Group 3 (Figure 2a). Th lymphocytes decreased following the severity of COVID disease (Figure 2c). B lymphocytes progressively increased from Group 1 to Group 3 (Figure 2d). A significant increment in Group 3 was exhibited by NK lymphocytes (Figure 2e). 

### 2.2. The Activated T Lymphocytes and the Treg and T_R3-56_ Cells in the Patients Stratified on the Severity of COVID Disease

**Figure 3:**
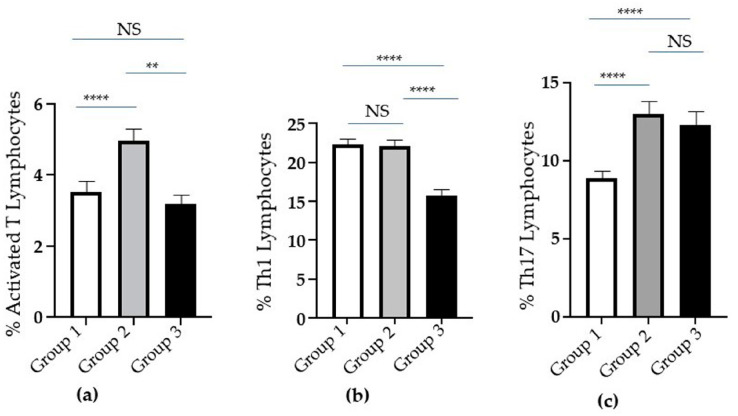
Analysis of activated T, Th1 and Th17 cells in groups of COVID-19 patients based on increasing severity. (**a**) The percentage of activated T, (**b**) Th1, and (**c**) Th17 lymphocytes in Groups 1 (white columns), 2 (grey columns), and 3 (black columns) of patients. Standard error (SE) bars are reported at the top of the columns. Statistical analysis (*Mann–Whitney test*) is reported: *p* ≤ 0.005 (**); *p* < 0.0001 (****); and not significant (NS).

Since T lymphocytes appeared to decrease with the severity of clinical conditions (from Group 1 to Group 3), with an increase in CTLs in Group 3, we assessed the activation state of T lymphocytes by evaluating the Human Leukocyte Antigen (HLA)-DR expression on their cell surface (see Section 4) in the three groups. Intriguingly, the percentage of activated T lymphocytes significantly increased in Group 2 when compared to Group 1 (Figure 3a). Conversely, the percentage of the same cells in Group 3 was lower than in Group 2 and appeared similar to that in Group 1 (Figure 3a).

In addition, the percentage of Th1 cells was reduced in Group 3, when compared to Groups 1 and 2 (Figure 3b). Interestingly, we observed an increase in B cells in the same group (Figure 2d).

Th17 cell percentage significantly increased in Groups 2 and 3 when compared to Group 1 (Figure 3c).

**Figure 4:**
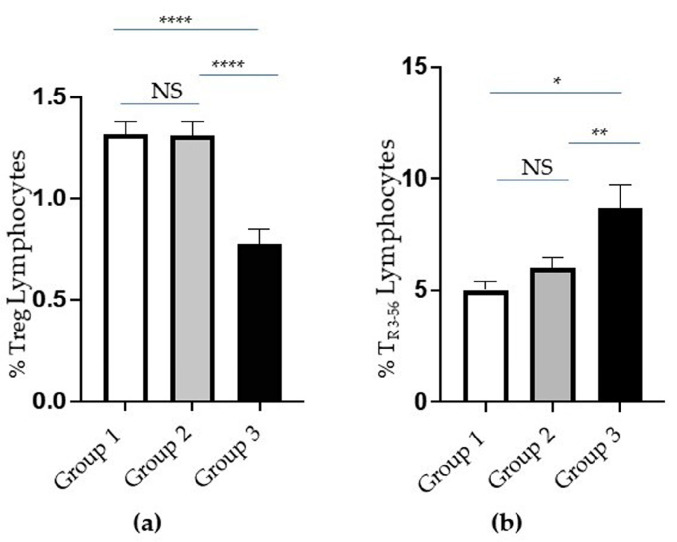
Analysis of T regulatory (Treg) and T_R3-56_ cells in groups of COVID-19 patients based on increasing severity. (**a**) The percentage of Treg and (**b**) T_R3-56_ lymphocytes in Groups 1 (white columns), 2 (grey columns), and 3 (black columns) of patients. Standard error (SE) bars are reported at the top of the columns. Statistical analysis (*Mann–Whitney test*) is reported: *p* ≤ 0.05 (*); *p* ≤ 0.005 (**); *p* < 0.0001 (****) and not significant (NS).

Notably, a significant reduction in the percentage of Treg cells is evident in Group 3 (Figure 4a). Conversely, the percentage of T_R3-56_ was significantly increased in Group 3, which expressed more severe clinical conditions (Figure 4b).

### 2.3. The Cytokines in the Patients Stratified on the Severity of COVID Disease

We analyzed the serum cytokine concentration in the three groups of patients (Table 1). Intriguingly, the TNF-α was significantly different between Group 1 and Group 2 (*p* = 0.0011) and between Group 1 and Group 3 (*p* = 0.0075). No statistic difference was observed between Group 2 and Group 3 (Table 1). In addition, no statistic differences were observed in IL-6, IL-17, and IL-10 concentrations between groups of patients (Table 1).

### 2.4. The Patients Stratified on the Basis of a Cut-Off Calculated on the T_R3-56_ Cell Distribution

Given that the percentage of T_R3-56_ cells increased in patients with more severe clinical conditions, while the percentage of Treg cells appears reduced, we focused our attention on the T_R3-56_ regulatory level in our patient cohort.

In this regard, the percentage of T_R3-56_ cells in the enrolled patients ranged widely from 0.3 to 20.5. The mean percentage value was 6.3, with a standard deviation (SD) of 4.5 and a SE of 0.7. Therefore, it is a distribution with the percentage values spread over a very wide range. To identify the more relevant existing correlations between T_R3-56_ lymphocytes and the cells and molecules involved in the antiviral response, we arbitrarily focused on those patients whose percentage value was higher than the 75th percentile (8.2%) and above the mean + 3 × SE (8.4%).

Adopting this criterion, we applied a cut-off of 8.4% to stratify the patients, resulting in a small group of patients (n = 24), with very high levels of T_R3-56_ cells (TR3-56^High^ Group).

Interestingly, this group is predominantly comprised of individuals from Group 2 (n = 8) and Group 3 (n = 14).

This finding supports the increase in CTLs observed in patients with more severe clinical conditions (Figure 2b) and the corresponding rise in T_R3-56_ cells in Group 3 (Figure 4b). Additionally, we observed a positive correlation between the percentage of T_R3-56_ cells and immune effector cells: CTLs and NK cells (Table 2). In addition, the percentage of T_R3-56_ negatively correlates to the monocytes in TR3-56^High^ Group (Table 2).

Notably, the percentage of T_R3-56_ positively correlates to the IL-17A production (Table 2).

No correlations were observed between T_R3-56_ and the other cells (lymphocytes, neutrophils, T, Th, Th1, Th17, Treg, and B cells) and TNF-α (Table 2).

## 3. Discussion

The current study aimed to investigate the presence of T_R3-56_ cells [33] during the antiviral inflammatory response in COVID-19. By elucidating the role of these cells, research could expand on previous findings regarding CD3+CD56+ T cells in SARS-CoV-2 infections [62].

Our current analysis reveals that the overall lymphocyte population among WBCs was significantly lower in Groups 2 and 3 than in Group 1. Such evidence emphasizes that the trend was consistent across the entire cohort, regardless of treatment status.

A significant increment in Group 3 patients is exhibited by NK lymphocytes. This occurrence confirms that these effectors are involved in identifying and eliminating virus-infected cells in COVID-19 patients [9,10,11,12,13].

Additionally, Groups 2 and 3 exhibited reduced percentages of T cells.

Conversely, a significant increase in CTLs was revealed in Group 3, highlighting an active effort by the immune system to eliminate the SARS-CoV-2-infected cells. B lymphocytes progressively increased from Group 1 to Group 3, suggesting that an active humoral immune response against SARS-CoV-2 is aimed at neutralizing the virus [9,10,11,12,13,27]. Notably, the increase in both T cells and B cells correlated with disease severity in our cohort of patients.

Furthermore, Group 3 exhibited a reduced percentage of Th1 cells [9,10,11,12,13,18].

The percentage of Th17 cells [9,10,11,12,13,25,26] significantly increased in Groups 2 and 3 when compared to Group 1. This increase may signify the onset of chronic inflammatory conditions in patients at the most severe stage of COVID-19 [9,10,11,12,13,25,26].

Although T lymphocytes decreased with the severity of clinical conditions (from Group 1 to Group 3), they were accompanied by an increase in CTLs in Group 3. Therefore, we evaluated the activation status of T lymphocytes across the three groups. Interestingly, the percentage of activated T lymphocytes significantly increased in Group 2 compared to Group 1. Conversely, in Group 3, the percentage of activated T lymphocytes was lower than in Group 2 and similar to that in Group 1.

We investigated the presence of T lymphocytes involved in regulating immune responses, which could explain the observed reduction in lymphocyte activation status. In this context, it is noteworthy that the percentage of Treg cells [34,35,36] exhibits a significant decrease in Group 3.

The T_R3-56_ subset could provide novel insights, distinct from the cytotoxic role typically attributed to the CD3+ CD56+ T cell population in several diseases [37,38,39,40,41,42,43,44,45,46,47,48,49,50,51,52,53,54,55,56] and in SARS-CoV-2 infection [62,63].

Notably, the increased presence of T_R3-56_ cells in Group 3 may suggest a compensatory response to heightened inflammation and immune activation driven by the observed increases in CTLs, B cells, and Th17 cells during severe phases in our patient cohort.

We analyzed serum cytokine concentration in the three groups of patients. Interestingly, TNF-α was significantly elevated in Groups 2 and 3. However, no statistical differences were observed in the levels of other cytokines between these patient groups. This finding suggests a potential role for TNF-α in influencing the severity of clinical conditions within these groups.

The concomitant increase in T_R3-56_ cells along with the rise in CTLs, B cells, Th17 lymphocytes, and TNF-α suggests an overall activation of the immune system. T_R3-56_ cells may play a regulatory role in balancing the immune response to prevent the excessive immune reactions, hyper-inflammation, and tissue damage observed in severe cases of COVID-19.

In patients not receiving therapy and belonging to the TR3-56^High^ Group, we observed that higher percentages of T_R3-56_ correlate with elevated levels of CTLs and NK cells.

In addition, there is a positive correlation between the percentage of T_R3-56_ cells and IL-17 levels in the TR3-56^High^ Group. IL-17 is produced by various immune cells, including Th17 cells, γδ T cells, natural killer T cells, and innate lymphoid cells [9,10,11,12,13,25,26]. No correlations were found between the other cell types and cytokines in the TR3-56^High^ Group.

The exacerbated immune response in COVID-19 necessarily involves an uncontrolled engagement of immune effectors and the release of pro-inflammatory molecules [1,2,3,4,5,6,7,9,10,11,12,13,17,19,25,26,32]. In this scenario, it is plausible to consider the observed increase in T_R3-56_ cells in our cohort of patients as an attempt to mitigate the exacerbated immune response. Such occurrence points to the regulatory ability of T_R3-56_ cells in COVID-19, as described in other pathological conditions [57,58,61].

Our findings highlight the need to further investigate the functional significance of T_R3-56_ cells in the context of SARS-CoV-2 infection and other viral diseases. While our study provides preliminary evidence of a potential correlation between T_R3-56_ cells and disease severity, additional research is necessary to fully understand the mechanisms behind this association and to explore the therapeutic implications.

## 4. Materials and Methods

### 4.1. Patients

All patients were clinically classified upon hospitalization according to the WHO classification [65]. Briefly, COVID infection was diagnosed using molecular analysis (RT-PCR) on nasopharyngeal swabs. Patients were classified according to the WHO ordinal scale, which categorizes their condition into Groups 1 to 7: (1) not hospitalized with normal activities; (2) not hospitalized but unable to resume normal activities; (3) hospitalized without supplemental oxygen; (4) hospitalized with supplemental oxygen; (5) hospitalized with high-flow oxygen, non-invasive ventilation, or both; (6) hospitalized with extracorporeal membrane oxygenation (ECMO), invasive ventilation, or both; and (7) death [65]. The study analyzed a cohort of 106 hospitalized COVID-19 patients with varying clinical severity, classified according to WHO severity categories 3 to 7. To ensure a robust retrospective analysis, we divided the patients into three groups: Group 1 (WHO Group 3), Group 2 (WHO Group 4), and Group 3 (WHO Groups 5, 6, and 7), following previously established methods [20,64]. This classification was based on the oxygen therapy requirements, reflecting the severity of the clinical condition of patients: Group 1 included patients not requiring supplemental oxygen (n = 22, 12 males and 10 females. Age range of 33-78, with a mean age of 56 years); Group 2 included those receiving supplemental oxygen (n = 60, 28 males and 32 females. Age range of 26–91, with a mean age of 62 years); Group 3 comprised patients needing high-flow oxygen, invasive ventilation, or ECMO (n = 24, 18 males and 6 females. Age range of 32–97, with a mean age of 69 years). In Group 3, 8 males died (age range of 63–95, with a mean age of 81 years). The number of recruited patients from the first COVID 19 wave was similar to that in the second wave (50 vs. 56), as is the distribution of patients from the first and second waves across Groups 1–3. To ensure that the patient population was not influenced by treatments, in the current retrospective evaluation we exclusively selected the data analysis of those hospitalized patients who did not previously undergo anti-inflammatory steroid therapy or azithromycin [66]. None of the patients received anti-SARS-CoV-2 vaccination prior to hospitalization.

Patients were admitted to the Section of Infectious Diseases of the University Federico II (Naples, Italy) during the first and second waves of the pandemic (2020–2021) [64]. Whole blood samples were collected at admission and after one week of hospitalization in tubes containing EDTA or free from anticoagulant and then immediately analyzed by flow cytometry. Serum samples were separated from blood cells after the collection.

Ethical approval for the study was obtained from the Ethical Committee of the University Federico II of Naples (protocol code 138/20, 14 April 2020). The study was performed in accordance with the Declaration of Helsinki. Informed consent was obtained from all participants.

### 4.2. Flow Cytometry

Immunophenotyping analysis was performed by multicolour flow cytometry, as described [20]. Briefly, CD45 was used to gate the viable lymphocyte cells. From this gate, CD3+ CD4+ cells were identified as Th, while CD3+ CD8+ as CTLs. Among Th, Th1 and Th17 were distinguished by specific surface markers, i.e., CXCR3 and CCR6, respectively. Moreover, human leukocyte antigen DR (HLA-DR) molecules were used as activation markers expressed on activated T lymphocytes. CD3 and CD45, and CD56 and CD19 were used to T (CD3+ CD45+), NK (CD45+ CD3- CD56+), and B (CD45+ CD19+) cell distribution for each patient. Treg cells were identified as CD3+ CD4+ CD25^High^ CD127^low^, and T_R3-56_ as CD3+ CD56+, as described [57].

### 4.3. Serum Cytokine Analysis

Serum IL-17A, IL-6, IL-10 and TNF-α levels were analyzed using human-specific enzyme-linked immunosorbent assay (ELISA) Max™ Set Deluxe kits (BioLegend, Inc., San Diego, CA, USA), as described [20]. The concentration values (pg/mL) of each cytokine were obtained by interpolating the absorbance values on the respective calibration curve.

### 4.4. Statistics

The statistical analysis was performed using the *Mann–Whitney test* to compare the differences between Group 1 and Group 2, Group 1 and Group 3, and Group 2 and Group 3. The correlations between variables were evaluated by Spearman’s rank-order correlation and Spearman’s rank correlation coefficient (rs) was calculated. Statistical analysis and graphics were performed by Prism 9, GraphPad Inc. (San Diego, CA, USA). *p* values < 0.05 were considered as significant.

## 5. Conclusions

Our findings offer valuable insights into the complex immunological dynamics of SARS-CoV-2 infection and underscore the potential role of T_R3-56_ cells as a remarkable component in the immune response against SARS-CoV-2.

Our findings might suggest the regulatory ability of T_R3-56_ cells in COVID-19. However, these cells could also be involved in cytotoxic and antiviral secretory functions, potentially serving as effector cells in this context, as suggested by other studies on CD3+ CD56+ T cells [37,38,39,40,41,42,43,44,45,46,47,48,49,50,51,52,53,54,55,56]. It is reasonable to hypothesize that T_R3-56_ cells might adapt their regulatory functions and exhibit additional effector roles in specific contexts, such as during infections or inflammations.

This intriguing hypothesis, which suggests the remarkable plasticity of the immune system [66,67], requires support from studies demonstrating this mechanism.

In this regard, studies on Tregs also suggested a versatile role: beyond their traditional function of suppressing immune responses to prevent autoimmune diseases and maintain immune balance, Tregs were found to exhibit dynamic functionalities [68,69]. Tregs also demonstrate cytotoxic activity against tumor cells through granzyme-dependent mechanisms [70]. This newly described ability enables them to directly target and eliminate tumor cells, which contrasts with their conventional role as immune suppressors. Moreover, Tregs can interact with non-immune cells and reside in non-lymphoid tissues, where they perform non-immunological functions primarily related to tissue repair and organ homeostasis [36].

Similarly, CD3+ CD56+ T cell subtypes, including the proposed T_R3-56_ cells, may play a dynamic role in the plasticity of the immune response [44,45,46,47,48,49,50,52,53,54,55,56]. These cells could adapt to different peripheral tissue environments, where they may exhibit both significant suppressive effects on effector lymphocytes and engage in complementary effector functions that enhance immune responses. Therefore, considering both perspectives, our hypothesis is that T_R3-56_ cells may play a dual role in the immune response to SARS-CoV-2 infection. On one hand, their regulatory function can help mitigate excessive inflammation and tissue damage, thereby contributing to immune homeostasis and facilitating tissue repair processes. On the other hand, the heightened presence of T_R3-56_ cells in severe cases of COVID-19 may also reflect a broader immune response aimed at contrasting the viral infection.

Although this retrospective study may have limited clinical applicability due to the numerous mutations in SARS-CoV-2 and the evolving nature of COVID-19 since the first and second waves, its primary goal is to enhance our understanding of T_R3-56_ cells. The study aims to elucidate their potential role in viral infections and propose their use as biomarkers. Moving forward, expanding our knowledge of T_R3-56_ cells, as well as CD3+ CD56+ T cells and their interactions with the immune system, could facilitate the development of targeted therapies for managing infections and other diseases where these cells are critically involved.

### Study Limitations

Being a retrospective analysis, our study did not evaluate the functional effects of T_R3-56_ cells, which limits our ability to assess their potential regulatory capacity or effector functions. Additionally, the study did not compare the data with a control group of healthy and/or pre-pandemic subjects, as the focus was on comparing patients with varying degrees of disease severity. No correlation was analyzed based on gender and age of the subjects in Groups 1–3. No evaluation was conducted on the SARS-CoV-2 strains infecting the patients. Comparative analysis with other viral infections beyond SARS-CoV-2 was not performed. Finally, the study did not include a longitudinal assessment of the patients, as all of them, following hospitalization, underwent therapies capable of altering the immune response.

## Figures and Tables

**Table 1:** Serum cytokine concentrations in Groups of COVID-19 patients based on increasing severity *.

	Group 1	Group 2	Group 3	
	Mean ± SE	Mean ± SE	Mean ± SE	Mann-Whitney (*p* Value)
TNF-α (pg/mL)	3.07 ± 0.04	9.45 ± 2.37	4.30 ± 0.61	**0.0011** group 1 vs. group 2 **0.0075** group 1 vs. group 3 NS group 2 vs. group 3
IL-6 (pg/mL)	49.61 ± 6.24	170 ± 50.71	52.77 ± 13.74	NS group 1 vs. group 2 NS group 1 vs. group 3 NS group 2 vs. group 3
IL-17 (pg/mL)	3.34 ± 0.27	4.86 ± 0.91	3.44 ± 0.61	NS group 1 vs. group 2 NS group 1 vs. group 3 NS group 2 vs. group 3
IL-10 (pg/mL)	7.31 ± 0.45	10.71 ± 1.93	11.65 ± 3.70	NS group 1 vs. group 2 NS group 1 vs. group 3 NS group 2 vs. group 3

* ELISA serum concentrations are reported. Significative values are reported in bold. Mean ± standard error (SE) and *p* value are reported.

**Table 2:** T_R3-56_ cells positively correlate with Interleukin (IL)-17, Natural Killer (NK), and Cytotoxic T cells (CTLs) in the TR3-56^High^ Group of patients *.

% T_R3-56_ Lymphocytes		
	Slope	*p* Value
TNF-α (pg/mL)	0.1336	0.6730
IL-17 (pg/mL)	0.6786	0.0106
% Lymphocytes	−0.3719	0.0735
% Monocytes	−0.6431	0.0007
% Neutrophils	0.2351	0.2688
% T lymphocytes	−0.1228	0.5675
% B lymphocytes	−0.3902	0.0594
% NK lymphocytes	0.4456	0.0291
% CTLs	0.4507	0.0271
% Th lymphocytes	−0.3846	0.0635
% Th1 lymphocytes	0.2662	0.2086
% Th17 lymphocytes	−0.1446	0.5001
% Treg lymphocytes	−0.2540	0.2311

* Spearman correlation is reported: r = slope; p = *p* value. The Spearman correlation coefficients range from −1 to +1. The sign of the coefficient (*r)* indicates whether it is a positive or negative monotonic relationship. A positive correlation means that as one variable increases, the other variable tends to increase as well. A negative correlation means that as one variable increases, the other tends to decrease. Values closer to −1 or +1 represent stronger relationships compared to values closer to zero. Significative values (*p*) are reported in bold.

## Data Availability

The data presented in this study are available on request from the corresponding author. The data are not publicly available due to the privacy.
